# Comparison between DNA Detection in Trigeminal Nerve Ganglia and Serology to Detect Cattle Infected with Bovine Herpesviruses Types 1 and 5

**DOI:** 10.1371/journal.pone.0155941

**Published:** 2016-05-25

**Authors:** Rodrigo Puentes, Fabrício Souza Campos, Agustin Furtado, Fabrício Dias Torres, Ana Cláudia Franco, Jacqueline Maisonnave, Paulo Michel Roehe

**Affiliations:** 1 Departamento de Ciencias Microbiológicas, Área de Inmunología, Facultad de Veterinaria, Universidad de la República Oriental del Uruguay (UdelaR), Montevideo, Uruguay; 2 Laboratório de Microbiologia Veterinária, Faculdade de Agronomia e Medicina Veterinária, Universidade de Brasília (UnB), Distrito Federal (DF), Brazil; 3 Laboratório de Virologia, Departamento de Microbiologia, Imunologia e Parasitologia, Instituto de Ciências Básicas da Saúde, Universidade Federal do Rio Grande do Sul (UFRGS), Porto Alegre, Brazil; The University of Melbourne, AUSTRALIA

## Abstract

Bovine herpesviruses (BoHVs) types 1 (BoHV-1) and 5 (BoHV-5) are alphaherpesviruses of major importance to the bovine production chain. Such viruses are capable of establishing latent infections in neuronal tissues. Infected animals tend to develop a serological response to infection; however, such response—usually investigated by antibody assays in serum—may eventually not be detected in laboratory assays. Nevertheless, serological tests such as virus neutralization (VN) and various enzyme-linked immunosorbent assays (ELISAs) are widely employed to check individual or herd status of BoHV infections. The correlation between detection of antibodies and the presence of viral nucleic acids as indicatives of infection in infected cattle has not been deeply examined. In order to investigate such correlation, 248 bovine serum samples were tested by VN to BoHV-1 and BoHV-5, as well as in a widely employed (though not type-differential) gB ELISA (IDEXX IBR gB X2 Ab Test) in search for antibodies to BoHVs. Immediately after blood withdrawal, cattle were slaughtered and trigeminal ganglia (TG) excised for DNA extraction and viral nucleic acid detection (NAD) by nested PCR. Neutralizing antibodies to BoHV-1 and/or BoHV-5 were detected in 44.8% (111/248) of sera, whereas the gB ELISA detected antibodies in 51.2% (127/248) of the samples. However, genomes of either BoHV-1, BoHV-5, or both, were detected in TGs of 85.9% (213/248) of the animals. These findings reveal that the assays designed to detect antibodies to BoHV-1 and/or BoHV-5 employed here may fail to detect a significant number of latently infected animals (in this study, 35.7%). From such data, it is clear that antibody assays are poorly correlated with detection of viral genomes in BoHV-1 and BoHV-5-infected animals.

## Introduction

Bovine herpesviruses type 1 (BoHV-1) and type 5 (BoHV-5) are members of the order *Herpesvirales*, family *Herpesviridae*, subfamily *Alphaherpesvirinae*, genus *Varicellovirus* [1]. Both are economically important veterinary pathogens and, like other alphaherpesviruses, are typically recognized for its capacity to establish latent infections in neuronal ganglia following primary infection [2, 3, 4].

BoHV-1 is the causal agent of a number of clinical conditions, including infectious bovine rhinotracheitis (IBR), infectious pustular vulvovaginitis (IPV), abortion and transient infertility [5,6]. When present, clinical signs of infection are usually mild and, despite the often high morbidity, mortality rates are usually low [7]. Following a pattern common to herpesviruses, the number of infected animals tends to be significantly higher than those displaying clinically apparent disease [4]. The closely related BoHV-5, on its turn, is the major causative agent of bovine herpetic encephalitis/meningoencephalitis, although eventually it has also been recovered from the genital and respiratory tracts of cattle [3, 7]. As with BoHV-1, the number of BoHV-5-latently infected animals, as detected by PCR in neuronal tissues, surpasses by far those which display clinical disease [8, 9]. However, BoHV-5 infections are more drastic: when signs of disease are evident, it is usually fatal [10, 11, 12].

In countries of the Northern hemisphere, although BoHV-1 infections are widespread, BoHV-5 is not a major concern. In some of those countries, the relatively low prevalence of BoHV-1 contributed to the successful implementation of BoHV-1 eradication schemes, essentially based on repeated serological testing and elimination of positive animals. To date, six European countries have achieved BoHV-1 eradication [1], but the infection remains endemic in most cattle producing countries in America, Europe and Asia [13, 14, 15].

In those countries where BoHV-1 has been eliminated, the absence of concurrent BoHV-5 infections certainly contributed to the success of eradication programs, since this might bring additional problems to the identification of infected animals [11]. Detection of BoHV-5 has rarely been reported in countries in the Northern hemisphere [11, 16]. However, BoHV-5 is becoming a major concern in the Southern hemisphere, particularly in Brazil, Argentina and Uruguay [17], where its prevalence as determined on occasions by serological testing [18, 19] or by detection of viral genomes in samples of slaughtered cattle [8, 9], seems quite significant, in those studies ranging from 29,2% in dairy cattle [18] to 87% in beef cattle [8]. In view of this, BoHV control or eradication strategies in such countries must contemplate infections with these two viruses.

Identification of infected animals is pivotal to contingency measures. However, BoHV-infected cattle may display little or no detectable antibody responses; yet serological tests reveal fluctuations in antibody titers in infected animals along time [20]. Animals may on occasions be reported as seronegative despite being infected [21]. Like all alphaherpesviruses, BoHV-1 and BoHV-5 establish latent infections, what is equivalent to state that animals become potential shedders of such viruses. Latently infected animals usually harbor viral genomes as episomes in sensory neural ganglia, particularly the trigeminal ganglia (TG), the most recognized site for BoHV-1 and BoHV-5 latency [3, 22].

In order to investigate the correlation between detection of antibodies in serological assays and nucleic acid detection (NAD) to identify BoHV-1 and BoHV-5-infected cattle, serum samples of cattle destined to slaughtering were tested for antibodies to BoHV-1 and BoHV-5 by virus neutralization and by a widely employed, commercially available gB ELISA. The results of these serological assays were compared to NAD of viral genomes by nested PCR (nPCR) in TG tissues.

## Materials and Methods

### Collection of samples at slaughtering

Samples were collected in abattoirs in the state of Rio Grande do Sul (Brazil, geographic coordinates: 31°46'18.3"S 52°24'59.1"W) (n = 200) and in the province of Minas, Lavalleja (Uruguay, geographic coordinates: 34°21'18.2"S 55°13'37.0"W) (n = 48). Blood samples and trigeminal ganglia (TG) were collected at the time of slaughtering from randomly chosen adult cattle (3 to 6 years old) of either the Hereford breed or crossbreds, of both genders, though most (~70%) were cows. Blood samples were processed and sera stored at– 20°C.

Trigeminal ganglia (TG) were collected in pairs as described [8, 9], stored individually in 6-well plates and transported to the laboratory under refrigeration. At the time of processing, the TG were fragmented in several pieces, divided and stored in 24-well plates and frozen at -80°C. All samples were carefully handled to avoid cross-contamination.

### Cell cultures and viruses

Madin-Darby bovine kidney (MDBK, originally ATCC- CCL22) cells were used to propagate BoHV-1 and BoHV-5 used as challenge viruses in neutralization assays. Cells were multiplied and maintained following standard methods in Eagle’s minimal essential medium (EMEM, Gibco) supplemented with 2–10% fetal bovine serum (SFB, Gibco) and antibiotics [10 IU/ml penicillin, 10 mg/ml streptomycin (Cultilab); 2 mg/ml amphotericin B (Cristalia)]. The BoHV-1 used in this study was the strain Cooper [23]. The BoHV-5 used throughout was strain EVI 88/95 [24].

### Virus neutralization tests (VN)

Neutralizing antibodies to BoHV-1 and BoHV-5 were assayed in 24-hour virus/serum incubation period, as recommended for international trade [12]. Briefly, sera were inactivated by 30 min incubation at 56°C. Twofold serum dilutions were prepared in 50 μl volumes of tissue culture medium and added to 96-well, sterile tissue culture plates, in four wells per dilution. Subsequently, serum dilutions were mixed with 50 μl of a suspension containing 100 TCID_50_ (50% tissue culture infectious dose) of either BoHV-1 (Cooper) or BoHV-5 (EVI 88/95). Strong positive control sera, weak positive control sera, and negative sera were included in each batch of tests. After a 24 h incubation at 37°C, 100 μl of a cell suspension containing 3–4 x 10^4^ cells were added to each well and plates incubated at 37°C in a 5% CO_2_ incubator for up to five days. Titers were determined with basis on the detection of cytopathic effect and calculated by the method of Reed and Muench [25].

### gB ELISA

The detection of anti-BoHV antibodies in sera was performed with a commercially available indirect gB ELISA (IDEXX IBR gB X3 Ab Test; HerdChek, IDEXX Laboratories). The test was performed according to the manufacturer’s instructions and results read at an optical density of 450 nm in a spectrophotometer.

### Viral nucleic acid detection (NAD)

Viral NAD was performed in total DNA extracted from approximately 50 mg of tissues of each TG as described previously [26]. A PCR targeting a region of circa 570 base pairs (bp) on the *UL44* gene (the gC coding gene) was performed to detect simultaneously BoHV-1 and BoHV-5 [24]. Type differentiation was achieved with a nested PCR (nPCR) performed with the products of the first reaction [8]. In the nPCR, primers targeting an internal region of BoHV-1 *UL44* gene were designed to give rise to a 161 bp product, whereas BoHV-5-specific primers target a 236 bp-long amplicon. To avoid contamination with PCR products, separate rooms were used for extractions of DNA from ganglia, to prepare PCR buffers, and to examine PCR products. Filter tips were used throughout; work benches were decontaminated with UV light and negative controls were included in every five reactions.

### Data analysis

Comparative sensitivity, specificity, positive (PPV) and negative predictive values (NPV), accuracy and Kappa indices were calculated using the Data Analysis Supplement for Excel^TM^ (Office System 2010 for Windows^TM^, Microsoft Corp). In order to provide a better visualization of results, serological and NAD assays were plotted in a Venn diagram [27].

## Results and Discussion

The results of all tests performed in the present study are summarized in Table 1 and detailed in Table 2. Neutralizing antibodies to BoHV-1 and/or BoHV-5 were detected in 111/248 (44.8%) of the samples examined. Neutralizing antibody-positive samples were discriminated as follows: 13.5% of the 111 VN-positive samples reacted to BoHV-1 only, whereas 9% of those reacted to BoHV-5 only; 77.5% of the 111 VN-positive sera had cross reacting neutralizing antibodies to both virus types.

**Table 1:** Detection of antibodies to BoHV-1 and / or BoHV-5 in serum [virus neutralization (VN) and gB ELISA] and nucleic acid detection (NAD) in trigeminal ganglia (TG) of slaughtered cattle (n = 248).

	BoHV-1^#^	BoHV-5^#^	BoHV-1 and/or BoHV-5^#^	Positive/Total*
VN	15 (13.5%)	10 (9%)	86 (77.5%)	111 (44.8%)
gB ELISA	NA	NA	127 (100%)	127 (51.2%)
NAD	15 (7%)	37 (17.4%)	161 (75.6%)	213 (85.9%)†

# Percentage calculated on the total of positives for each technique.

* Percentage calculated on the total samples.

NA: not applicable, as the assay does not differentiate BoHV-1 from BoHV-5 antibodies.

† The difference to total number of sampled animals (n = 248) to VN, gB ELISA and NAD, is respectively, 137 (55.2%), 121 (48.8%) and 35 (14.1%).

**Table 2:** Comparison between results of: Virus neutralization (VN) and gB ELISA; VN and nucleic acid detection (NAD); NAD and gB ELISA. Sensitivity, specificity, negative (NPV) and positive (PPV) predictive values (CI = 95%) are shown.

Test	N° of samples with the following result				Sensitivity(%)	Specificity(%)	PPV(%)	NPV(%)	Accuracy(%)	Kappaindex(κ)
	True positive(TP)	False negative(FN)	False positive(FP)	True negative(TN)						
VN vs gB ELISA	96	31	15	106	0.75	0.87	0.86	0.77	0.81	0.63
VN vs NAD	104	109	7	28	0.48	0.80	0.93	0.20	0.53	0.13
gB ELISA vs NAD	122	91	5	30	0.57	0.85	0.96	0.24	0.61	0.21

Sensitivity = TP/(TP + FN). Specificity = TN/(FP + TN). PPV = TP/(TP + FP). NPV = TN/(TN + FN). TP is the number of samples with a true-positive result. FN is the number of samples with a false-negative result. TN is the number of samples with a true-negative result. FN the number of samples with a false-negative result. Accuracy = (TN + TP)/(TN+TP+FN+FP) = (Number of correct assessments)/Number of all assessments). Kappa index was measured to check the agreement between two diagnostic tests.

The gB ELISA (Table 1) detected antibodies to either BoHV-1 or BoHV-5 in 127/248 (51.2%) sera. As there is no commercially available gB ELISA specifically designed to detect antibodies to BoHV-5, this widely used test kit was employed. The gB ELISA has been exhaustively employed by others to detect BoHV-1 antibodies [21, 28]; however, previous studies on its capacity to detect BoHV-5 antibodies were rather limited [29] (Wellenberg et al., 2001).

The gB ELISA is aimed to detect antibodies to gB; as this protein bears 95.9% amino acid similarity [30] between BoHV-1 and BoHV-5, good sensitivity was expected from the gB ELISA in its capacity to detect not only BoHV-1 but also BoHV-5 anti-gB antibodies. Indeed, this was the case in the present study; the gB ELISA detected more positive samples (n = 127) than VN performed with either BoHV-1 or BoHV-5 as challenge viruses (n = 111).

Adding the positive results of VN and gB ELISA, the two serological tests detected antibodies in 142 (57.3%) out of the total 248 serum samples.

When examining the results of NAD assays on TGs of the serum sampled animals collected at slaughtering (Table 1), 213 out of the total 248 (85.9%) animals were found to contain genomes of either BoHV-1, BoHV-5, or both in its TGs. Of these, 7% contained only BoHV-1 DNA; 17.4% contained only BoHV-5 DNA, whereas 75.6% of the samples contained DNA of both viruses. Thus, the disagreement between positive results of serological tests and NAD was at the order of 28.6%. In other words, approximately 3 out of 10 viral DNA bearing (infected animals) were not detected by the serological tests employed here.

Contingency table were constructed to compare the assays with each other and to determine its relative efficiencies (Table 2). From such data, it is clear that NAD assay detected a greater number of infected animals than the serological tests. Taking the NAD assay as reference, the sensitivity of the VN was 48% with 80% specificity, whereas the gB ELISA, the sensitivity was 57%, with 85% specificity. The level of agreement between VN and gB ELISA was quite substantial (κ = 0.63). However, when comparing VN to NAD, a slight agreement (κ = 0.13) was detected. When the gB ELISA was compared to NAD, the level of agreement was fair (κ = 0.21).

**Fig 1:**
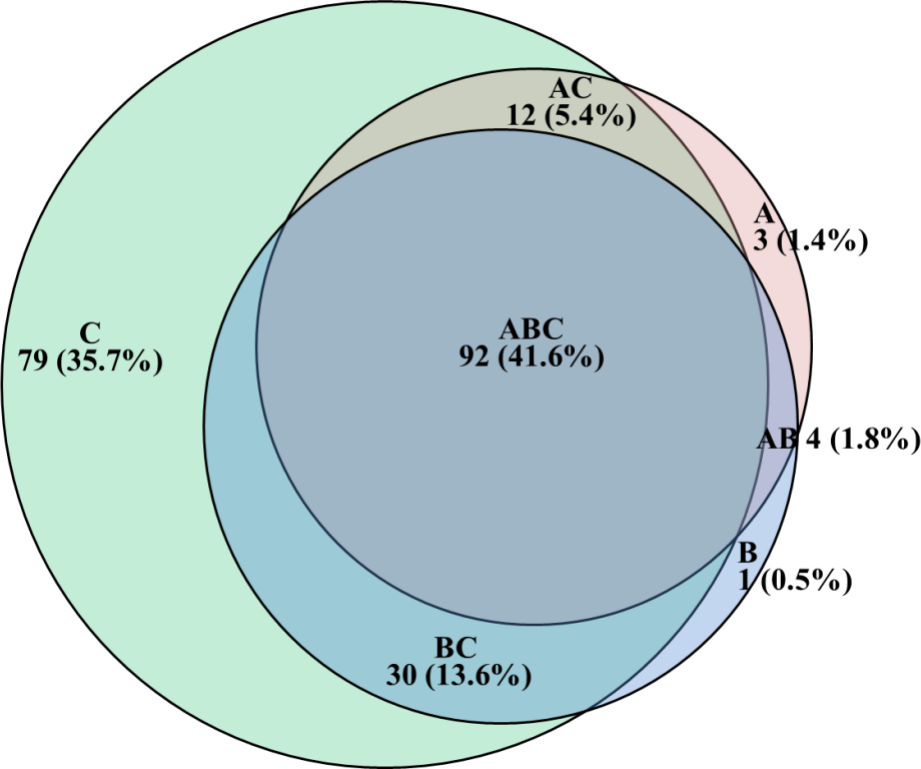
Venn diagram showing the distribution of samples which resulted positive in at least one of the assays performed (n = 221). (A) virus neutralization (VN); (B) gB ELISA; (C) Nucleic acid detection (NAD) by nPCR.

The combined results of serological assays and NAD were used to plot a Venn diagram [27] (Fig 1). The diagram allows a more discriminative visualization of the profile of reactivity of samples in each of the tests. The total number of positive samples in at least one of the assays was 221/248, corresponding to 89.1% of the animals sampled.

When comparing NAD to serological tests, it became evident that prevalence of BoHV-1 and BoHV-5 infections in the sampled population, as determined by antibody detection, was significantly lower than that revealed by NAD The gB ELISA detected 122/221 (55.2%) and VN detected 111/221 (50.2%) positive samples. Samples which reacted positively in the three assays corresponded to 41.6%. Thus, 35.7% of the samples were positive only by the NAD. Therefore, by serologic, 35.7% of the infected animals will be reported as uninfected, despite latently infected.

Obviously, serological assays, which are accepted as standards for international trade [12] are not designed to detect latently infected animals; antibodies in serum indicate that cattle were either vaccinated or infected at some time in the past. However, some seronegative animals—or animals whose antibody levels might be below the trough level of the serological assays employed here, may still be infected, as could be seen here, as evidenced by seronegative animals which nevertheless carry viral DNA.

Unfortunately, to date, there are no tests capable of detecting latently infected animals *in vivo;* NAD assays can only be performed on *post mortem* examinations. Therefore, until *in vivo* NAD assays become available, BoHV-1 or BoHV-5 incidence or prevalence will rely on serological tests, whose sensitivity was shown here to be significantly lower than NAD assays in its ability to detect infected animals.

Eight samples in this study did not react positively at NAD but resulted positive at VN (3 samples), at gB ELISA (1 sample) or at both serological assays (4 samples) (Fig 1). These probably correspond to uninfected calves which were vaccinated with inactivated vaccines. This is a speculation by the authors, since no vaccination history was available to substantiate such hypothesis. Nevertheless, it seems very likely that such samples were from uninfected cattle, vaccinated with inactivated vaccines. This combination might lead to seropositive cattle which were indeed not infected with either virus type. Serological “false positive” results may lead to important losses by condemning animals of economic or genetic value. In the present study, the NAD method employed might have missed genomes in TG tissues with less than 25 genome copies/200 ng of total ganglion DNA, which is the through level of the nPCR employed [8]. However, it seems likely that those eight samples were from vaccinated, uninfected animals. Unfortunately, it was not possible to trace back the vaccination history of the animals participating in the study; nevertheless, the authors believe that previous vaccination would be most likely to explain such findings. Therefore, until methods for detection of latent infections *in vivo* become available, such animals would be considered infected by serological assays, unless vaccinated with differential vaccines, which was certainly not the case in the present study, since no differential BoHV vaccines are licensed in the countries where samples were collected.

The findings reported here provide evidence that serological tests to BoHV-1 or BoHV-5 must be interpreted with caution, particularly when examining isolated serum samples, as in the present study. Under such conditions, a significant number of animals may be serologically non-reagents despite carrying viral genomes and, as such, being potential virus shedders. Animals may, at the time of sample collection, have low antibody levels which may have gone undetected at VN or at gB ELISA. Fluctuation in antibody titers along time is a recognized fact in herpesvirus infections [19, 20, 31, 32, 33, 34, 35]. It is likely that such fluctuation might be amongst the causes of at least some of the “false negative” serological results in the assays performed here.

The most relevant finding of this study is the fact that a significant number of animals potentially carrying viral genomes (35.7%) would not be detected by either of the serological assays employed here. This is highly relevant in that false negative animals might compromise attempts to control or eradicate such infections, which usually involve strategies based on the identification and removal of infected animals.

## Conclusion

The results presented here highlight the limitations of methods of detection of antibodies when compared to NAD in the identification of BoHV-1 and BoHV-5-infected animals. Detection of latently infected animals still relies on *post mortem* identification of viral genomes in tissues such as TG. Research on more sensitive serological methods as well as on methods to detect latently infected animals *in vivo* would bring a significant contribution to diagnosis, control and/or eradication of such infections.
